# Mouse Models of Cardiomyopathies Caused by Mutations in Troponin C

**DOI:** 10.3390/ijms241512349

**Published:** 2023-08-02

**Authors:** Svetlana B. Tikunova, Jenna Thuma, Jonathan P. Davis

**Affiliations:** Department of Physiology and Cell Biology, Dorothy M. Davis Heart and Lung Research Institute, College of Medicine, The Ohio State University, Columbus, OH 43210, USAdavis.812@osu.edu (J.P.D.)

**Keywords:** troponin, cardiomyopathy, cardiac, thin filament, contraction, relaxation, calcium sensitivity, kinetics

## Abstract

Cardiac muscle contraction is regulated via Ca^2+^ exchange with the hetero-trimeric troponin complex located on the thin filament. Binding of Ca^2+^ to cardiac troponin C, a Ca^2+^ sensing subunit within the troponin complex, results in a series of conformational re-arrangements among the thin filament components, leading to an increase in the formation of actomyosin cross-bridges and muscle contraction. Ultimately, a decline in intracellular Ca^2+^ leads to the dissociation of Ca^2+^ from troponin C, inhibiting cross-bridge cycling and initiating muscle relaxation. Therefore, troponin C plays a crucial role in the regulation of cardiac muscle contraction and relaxation. Naturally occurring and engineered mutations in troponin C can lead to altered interactions among components of the thin filament and to aberrant Ca^2+^ binding and exchange with the thin filament. Mutations in troponin C have been associated with various forms of cardiac disease, including hypertrophic, restrictive, dilated, and left ventricular noncompaction cardiomyopathies. Despite progress made to date, more information from human studies, biophysical characterizations, and animal models is required for a clearer understanding of disease drivers that lead to cardiomyopathies. The unique use of engineered cardiac troponin C with the L48Q mutation that had been thoroughly characterized and genetically introduced into mouse myocardium clearly demonstrates that Ca^2+^ sensitization in and of itself should not necessarily be considered a disease driver. This opens the door for small molecule and protein engineering strategies to help boost impaired systolic function. On the other hand, the engineered troponin C mutants (I61Q and D73N), genetically introduced into mouse myocardium, demonstrate that Ca^2+^ desensitization under basal conditions may be a driving factor for dilated cardiomyopathy. In addition to enhancing our knowledge of molecular mechanisms that trigger hypertrophy, dilation, morbidity, and mortality, these cardiomyopathy mouse models could be used to test novel treatment strategies for cardiovascular diseases. In this review, we will discuss (1) the various ways mutations in cardiac troponin C might lead to disease; (2) relevant data on mutations in cardiac troponin C linked to human disease, and (3) all currently existing mouse models containing cardiac troponin C mutations (disease-associated and engineered).

## 1. Introduction

The umbrella term “cardiomyopathy” refers to a diverse group of diseases affecting the ability of cardiac muscle to pump oxygenated blood to the rest of the body [1,2]. Cardiomyopathy can be “acquired” if it develops after birth due to infections, other health conditions, or environmental factors; or “genetic” if it is caused by a pathogenic gene variant [1,3,4]. It is possible that, in some cases, cardiomyopathy is triggered by a combination of acquired and genetic factors [4,5]. The three original sub-types of primary cardiomyopathy are hypertrophic (HCM), which occurs in 1:200–500 individuals [6,7,8]; dilated (DCM), which occurs in 1:500 individuals [9,10]; and less common restrictive (RCM), which only represents up to 5% of all cardiomyopathy cases [1,11]. Another uncommon cardiac disorder, left ventricular non-compaction cardiomyopathy (LVNC), was classified by the American Heart Association as a primary form of cardiomyopathy in 2006 [12,13]. Although researchers had hoped there might be a common molecular pathway associated with each type of cardiomyopathy (such as a change in Ca^2+^ sensitivity of force development), the disease drivers may be highly personalized [3].

In general, HCM is a disease of the cardiac muscle associated with the thickening of ventricular walls, most commonly occurring at the septum. Hallmark features of HCM include normal or even increased systolic function and diastolic dysfunction. HCM can be divided into obstructive, where thickened heart muscle reduces or blocks blood flow from the left ventricle to the aorta, and non-obstructive. HCM can present with a vast range of clinical phenotypes, with a majority of individuals remaining asymptomatic and not needing clinical interventions [14]. However, 30–40% of individuals with HCM experience adverse cardiovascular events, such as atrial fibrillation, ventricular tachycardia/fibrillation, stroke, heart failure, or sudden cardiac death [14]. In fact, sudden cardiac death can be the only presentation of HCM, especially in younger individuals. Some HCM patients are asymptomatic early in the progression of the disease but develop symptoms, such as shortness of breath, chest pains, and dizziness, over time. HCM is considered an inheritable condition, with ~40–60% of HCM cases associated with pathogenic variants in genes encoding sarcomeric proteins [14]. To date, more than 1400 mutations in at least 11 genes encoding sarcomeric proteins alone have been linked to HCM, with ~70% of sarcomeric pathogenic variants found either in myosin binding protein C or β-myosin heavy chain [14].

DCM is a disease of the cardiac muscle associated with left ventricular dilation, normal or thinned ventricular walls, and impaired systolic function [15]. Many patients with DCM also exhibit impaired diastolic function. Genetic mutations account for ~20–35% of DCM cases [16]. The DCM-linked genes encode a diverse group of proteins, including nuclear envelope, Z-disc, desmosome, RNA-binding, co-chaperone, cytoskeletal, and sarcomeric proteins [17].

RCM is a disease of cardiac muscle in which the ventricular muscle becomes abnormally rigid, resulting in the inability of ventricles to properly relax or fill with blood. Systolic function usually remains normal, at least early in the progression of the disease. Most cases of RCM are acquired, but genetic links are found in ~25–30% of cases. The majority of RCM-linked mutations have been found in genes encoding sarcomeric proteins [18].

LVNC is a very rare condition of genetic origin, characterized by excessive left ventricular trabeculation with deep intertrabeculae recesses. LVNC has been linked to mutations in genes encoding mitochondrial, cytoskeletal, structural, and sarcomeric proteins [19,20]. While some affected individuals remain asymptomatic, others experience adverse events such as life-threatening arrhythmias, thromboembolism, congestive heart failure, and sudden cardiac death.

Since all sorts of seemingly unrelated proteins and potential molecular mechanisms may lead to cardiomyopathies, it has been difficult to ascertain if a common or personalized mechanism is at play. It may be insightful to focus the spotlight on a single gene in which mutations have been linked to each of the cardiomyopathies, such as TNNC1.

## 2. Role of cTnC in the Regulation of Cardiac Muscle Contraction

Cardiac troponin C (cTnC), encoded by TNNC1, is a small sarcomeric protein that regulates the contraction of cardiac muscle via first sensing the Ca^2+^ signal and then initiating the cascade of events that leads to force production [21]. CTnC contains N- and C-globular domains, each containing two helix-loop-helix EF-hand Ca^2+^ binding motifs connected by a flexible helical linker. The Ca^2+^ binding EF-hand motifs are numbered I through IV, with helices flanking the loops labeled A-H. The N-terminal of cTnC contains an additional 14 residue N-helix, lacking in the closely related EF-hand Ca^2+^ binding protein calmodulin (CaM). The first EF-hand of cTnC is unable to bind Ca^2+^ with physiologically relevant affinity. Compared to the C-domain, the N-domain of cTnC has lower Ca^2+^ sensitivity and faster Ca^2+^ exchange rates [21,22]. The C-domain of cTnC also binds Mg^2+^ and remains occupied by either Ca^2+^ or Mg^2+^ even during diastole. Thus, the N-domain is thought to directly regulate muscle contraction via binding and release of Ca^2+^, while the C-domain is believed to play a structural role of anchoring cTnC onto the thin filament [21,22]. However, cardiomyopathy-linked mutations have been found in both the N- and C-domains, as well as the central helix, suggesting the importance of the entire protein in the proper regulatory function of cTnC [23].

## 3. Why Focus on cTnC

There may not be a simpler Ca^2+^-binding protein to link its Ca^2+^-binding properties to its in vivo physiological function than cTnC. Unlike CaM, which is known to bind and interact with hundreds of target proteins, many of which themselves strongly impact physiological outcomes [24,25], cTnC is found in cardiac myocytes only as a part of the cardiac troponin (cTn) complex, located primarily in the muscle lattice, but also in the nucleus of developing cardiac myocytes [26]. The cTn complex is stoichiometrically anchored on the thin filament (there is little to no free cTnC in cardiac myocytes) [22]. Although cTnC is capable of binding three Ca^2+^ ions per molecule, binding of a single Ca^2+^ ion to the second EF-hand of cTnC is thought to directly modulate cardiac muscle contraction (as opposed to binding of two Ca^2+^ ions to the N-domain of fast skeletal TnC and up to four Ca^2+^ ions to both N-and C-domains of CaM). A single mutation within the Ca^2+^-binding loop of the second Ca^2+^-binding EF-hand of cTnC completely abolishes the ability of Ca^2+^ to cause cardiac muscle contraction [27]. On the other hand, the formation of intra-molecular disulfide bonds between two native Cys residues (Cys35 and Cys84 (a mutation in the latter will be described below)) within cTnC causes maximal contraction even without Ca^2+^ [28]. Thus, modulating cTnC alone has the capability of completely turning off or on an otherwise normal cardiac muscle. Further, unlike almost every other cardiac muscle protein, there are no isoforms of cTnC in mammals to compensate for its function in the heart when lost since the only other homolog, fast skeletal TnC, is not expressed in cardiac myocytes. Homozygous knock-out of cTnC is embryonically lethal, whereas knock-out (or loss of complete function) of just one allele does not significantly affect cardiovascular function in mice (https://www.mousephenotype.org, accessed on 30 June 2023), indicating that cardiac muscle can be provided with sufficient cTnC to regulate muscle function from a single allele. More importantly, we know how to directly tune the Ca^2+^-binding properties of cTnC [29,30,31,32,33,34,35].

As would be expected, altering the Ca^2+^ binding affinity of cTnC shifts the Ca^2+^ sensitivity of actomyosin ATPase activity, force development, kinetics of contraction, and in vivo hemodynamics [21,36,37]. Although increasing the Ca^2+^ binding affinity of cTnC increases the Ca^2+^ sensitivity of force development, it does not necessarily increase the maximal force that the muscle can produce, whereas decreasing the Ca^2+^ binding affinity of cTnC can lead to the inability of Ca^2+^ to reach its maximal saturation of force [38,39]. Thus, cTnC is an ideal Ca^2+^-binding protein that can be methodically studied and manipulated to better understand the relationship between the biochemistry of Ca^2+^ binding with that of a physiological function.

## 4. Ca^2+^ Exchange Kinetics of cTnC

In vivo, the heart will be in a “steady-state” only once, death. We and others have observed that altering the Ca^2+^ binding properties of cTnC can have a substantial impact not only on the amount of force the muscle will produce for a given concentration of Ca^2+^ but also on the rates of both contraction (isometric and isotonic) and relaxation (isometric and isotonic) [38,39,40]. Thus, it is not only important to understand the overall Ca^2+^ sensitivity of cTnC but how quickly cTnC can transition between binding and releasing Ca^2+^. Not surprisingly, when Ca^2+^ sensitivity is discussed, it is widely accepted that the properties of cTnC must be at play in influencing the overall steady-state value. However, once kinetics are discussed, the vast majority of muscle researchers generally ignore cTnC and focus on how quickly Ca^2+^ itself is handled (the dynamics of the Ca^2+^ transient) or which isoform of myosin is present in the heart (there is a fast and a slow cardiac version of myosin in mammals) [41]. The tendency to not consider cTnC as an influencer of muscle contraction and relaxation kinetics is due to the false impression that cTnC is always in rapid equilibrium with a change in Ca^2+^ concentration. In other words, it is generally thought that the dynamics of the Ca^2+^ signal itself directly control how quickly cTnC binds Ca^2+^. Following this line of reasoning, the rate of Ca^2+^ binding to cTnC is assumed to be diffusion controlled, limited solely by how quickly Ca^2+^ can reach cTnC. It is not clear what precisely the rate of diffusion is within the sarcomeric lattice considering there is also fluid flow (advection currents) aiding the movement of solutes throughout the lattice [42]. Regardless, if the Ca^2+^ association is a fixed constant, then the Ca^2+^ sensitivity can only be modulated by altering how quickly Ca^2+^ dissociates from cTnC (K_d_ = K_off_/K_on_) [22]. The rate of Ca^2+^ dissociation from cTnC is generally thought to be orders of magnitude faster than the fall in the Ca^2+^ signal (but not for the C-domain of cTnC, which has always been accepted to have a “slow” Ca^2+^ dissociation rate) [29]. Thus, even though cTnC is accepted to modulate the overall Ca^2+^ sensitivity of cardiac muscle, it has not been accepted to influence the kinetics of muscle contraction or relaxation. We believe this inaccurate way of thinking about cTnC stems from the fact that the Ca^2+^ exchange kinetics of isolated cTnC (with no other proteins present in the solution) are very rapid [22]. However, cTnC does not function in muscle in isolation, and the addition of the other muscle proteins, especially cTnI, has a profound impact on how quickly (or more importantly, slowly) Ca^2+^ exchanges with cTnC [33,37,43]. We have demonstrated that the Ca^2+^ dissociation rate from cTnC in myofibrils (a biochemical preparation that contains the proper stoichiometry and geometric constraints of cTnC in muscle) is slow enough to potentially influence relaxation [41,44].

Contrary to dogma, one of the more surprising discoveries we made as we engineered cTnC was that cTnC could be sensitized, or desensitized, to Ca^2+^ by affecting its intrinsic rate of Ca^2+^ association. For instance, the single point mutation, L48Q, enhanced the Ca^2+^ association rate to isolated cTnC nearly an order of magnitude and more than five-fold when incorporated into the thin filament [32,33]. We have similarly observed that the Ca^2+^ sensitizing disease mutation L29Q in cTnC accelerates the rate of Ca^2+^ association with cTnC [45]. Furthermore, we have shown that disease-associated mutations in cTnT and cTnI also alter the apparent Ca^2+^ association rate to cTnC when incorporated on the thin filament [46]. It is becoming clear that a change in Ca^2+^ sensitivity cannot be assumed to be driven only by an equivalent change in the rate of Ca^2+^ dissociation from cTnC. By no means are we suggesting a change in the Ca^2+^ association rate is more significant to the setpoint of Ca^2+^ sensitivity than a potential change in the Ca^2+^ dissociation rate, only that it is not straightforward, or even possible, to accurately extract kinetic information from a steady-state curve. In fact, if one allows for the possibility that the rate of Ca^2+^ association can be modulated (either by design or disease), then there are at least five unique combinations of altering the association and dissociation rates that would lead to an overall increase in the measured Ca^2+^ sensitivity [47].

We and others have clearly demonstrated that engineered and disease-linked mutations in cTnC not only influence the rate of Ca^2+^ association to cTnC but also can strongly influence the rate of Ca^2+^ dissociation from cTnC [33,37,43,46]. Unfortunately, very few researchers attempt to tackle the kinetic properties of cTnC (or rates of muscle contraction and relaxation, for that matter), in part because dogma has suggested it does not matter, but also since the equipment and experimental setups to capture kinetic changes are much more complicated than those needed for steady-state measurements. Although we believe the kinetics of Ca^2+^ exchange with cTnC (or at least the rates of thin filament activation and deactivation) can have a major impact on the overall rates of cardiac muscle contraction and relaxation, it is beyond the scope of this review to dig deeper into this topic, and we refer the reader to several other review articles that focus deeper on this controversial topic [22,41,48]. When thinking about Ca^2+^ binding experiments, one must be cognizant if the measurement is an intrinsic property (the actual Ca^2+^ binding K_d_, association rate, or dissociation rate for cTnC under specific salt, temperature, and buffer conditions) or an apparent property (a measurement influenced by the intrinsic properties, but no longer equivalent to the intrinsic values). Some scenarios which result in the observer no longer measuring the intrinsic properties of cTnC but rather an apparent value are competitive interactions, multi-component reactions, or structural constraints which minimize reaction volumes. It is not always straightforward to know if one is observing an intrinsic property or an apparent property, even though this is essential when interpreting data and building models for how muscle functions [49].

## 5. What Can Go Wrong with cTnC Function?

**Figure 1:**
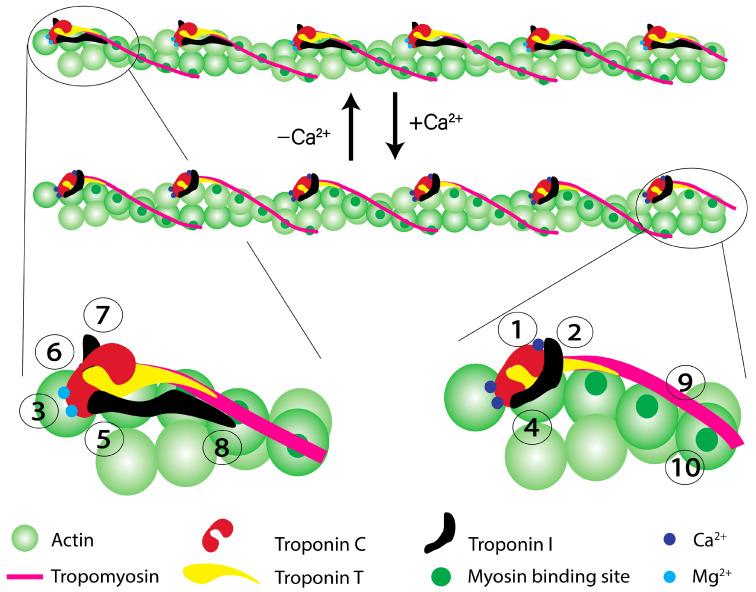
What Can Go Awry with cTnC? The figure shows a cartoon representation of the cardiac thin filament in the presence and absence of Ca^2+^ on the top and representative “zoom in” on the part of the thin filament in the two states. Overlayed on the amplified part of the thin filament are numbers 1 through 10, highlighting areas in the thin filament that could be disease driving as described in the text. The labeling scheme for the molecules is located at the bottom of the figure.

Figure 1 depicts a cartoon representation of the structural changes that occur within the thin filament in order to transition from a relaxed muscle at resting Ca^2+^ conditions to a contractile state after a rise in Ca^2+^ (keep in mind most Ca^2+^ transient measurements are that of free cytosolic Ca^2+^ (~1 µM), not the total Ca^2+^ released into the cytosol (100 to 200 µM as the total concentration of cTnC in cardiac myocyte is ~60 µM) [50]. It is not hard to envision that if cTnC does not bind Ca^2+^, or does so too strongly, then force output will be adversely affected (**point 1** in potential ways cTnC can go awry), considering the heart normally operates at ~30 to 50% of its capacity [51]. Furthermore, even under saturating Ca^2+^, the thin filament (in the absence of myosin) is not completely “turned on” [52,53]. One of the striking observations from numerous studies made on diseased myocardium is an observed change in the Ca^2+^ sensitivity of force production (keep in mind this is an apparent value as these measurements are not directly following Ca^2+^-binding to cTnC, nor necessarily reflecting a change in the intrinsic affinity of cTnC for Ca^2+^). Considering how rare cTnC mutations are in the general population [54], the vast majority of these Ca^2+^ sensitivity changes in cardiac disease occur in the presence of a “normal” cTnC, with disease-linked mutations in other proteins influencing the apparent Ca^2+^ binding affinity of cTnC. Thus, in addition to the intrinsic ability of cTnC to bind Ca^2+^, there are a plethora of interactions to and within the myofilaments and their local environment that can influence the apparent Ca^2+^ binding affinity of cTnC.

One of the biggest influences on the apparent Ca^2+^-binding properties of cTnC is the ability of cTnC to actually bind to the regulatory unit (switch peptide) of cTnI (**point 2**) [37]. There is evidence that some mutations in cTnC lead to an increased intrinsic affinity for the switch peptide of cTnI [55]. The apparent Ca^2+^ binding affinity of the regulatory N-domain of cTnC increases upon binding the switch peptide of cTnI in a dose-dependent manner [49]. Fascinatingly, the intrinsic affinity of the switch peptide for the Ca^2+^ bound regulatory N-domain of cTnC is quite low (K_d_ of less than 50 µM) for a one to one protein interaction that controls such an important functional output such as force production [49,56]. However, the switch peptide is not the only region of cTnI that cTnC binds. CTnC is essentially anchored to cTnI even at resting Ca^2+^ concentrations due to the fact that the C-domain of cTnC also binds Mg^2+^ with moderately high affinity that keeps cTnC bound and docked to the N-domain of cTnI (**point 3**—thus a mutation in the C-domain of cTnC may interfere with the cTn complex formation). If cTnC is not able to efficiently bind to the thin filament, that portion of the thin filament is effectively turned off (assuming a wild-type cTnC expressed from a non-mutated allele does not move into the vacant site). Furthermore, some cTnC mutations actually result in stronger binding of cTnC to the thin filament [23]. The anchoring of cTnC to the N-terminal of cTnI, in essence, helps to keep the switch peptide and the regulatory N-domain of cTnC always physically “tethered” together within a very small volume space giving this reaction a rather high “effective concentration” (**point 4**) [49]. There is some evidence that the regulatory N-domain of cTnC also interacts with cTnT, potentially influencing the effective concentration of the switch peptide [57].

Unlike fast skeletal TnC that can be rapidly and readily removed from the contractile apparatus by the simple chelation of both Ca^2+^ and Mg^2+^, cTnC is not so easily removed from the contractile apparatus of cardiac muscle and may have more contacts keeping cTnC in the cTn complex anchored to the thin filament [38,58]. The N-terminal extension of cTnI, which appears to wrap around the backside of the regulatory N-domain of cTnC, also helps to hold cTnC in a position to more readily bind and accept the switch peptide of cTnI (**point 5**) [59,60]. Additionally, cTnI interactions with the central helix of cTnC may also help to keep cTnC in a more accepting orientation to bind the switch peptide of cTnI (**point 6**) [61]. Thus, an emerging picture is that the regulatory interaction between cTnC and cTnI is influenced strongly by not only being tethered together, keeping the two proteins in very close proximity, but also being held in a particular orientation that also facilitates binding. Thus, the low intrinsic affinity of the switch peptide of cTnI for cTnC is overcome by not relying on diffusion to find one another since the two proteins are already structurally together. In this regard, muscle seems to be designed in such a way that none of its systems (electrical, Ca^2+^-handling, and mechanical) appear to rely entirely on the free energy of diffusion, which is slow and directionless [62,63,64].

There are a plethora of post-translational modifications to several of the myofilament proteins that can alter the apparent Ca^2+^-binding properties of cTnC [36,54]. The classic example occurs during the fight or flight response in which PKA activation phosphorylates two Ser residues on the N-terminal extension of cTnI that desensitizes cTnC to Ca^2+^ (**point 7**) [51,65,66,67]. Several kinases can phosphorylate several different Ser, Thr, and Tyr residues on the cTn complex alone that have the ability to either desensitize (i.e., PKA), sensitize (i.e., AMPK) or, depending on where the phosphorylation event occurs, sensitize and desensitize (i.e., PKC) thin filament activation and thus force production to Ca^2+^ [68,69,70]. Surprisingly, none of these post-translational modifications actually directly phosphorylate cTnC, but rather cTnI and cTnT, the partner proteins. The emerging picture is that these phosphorylation events either enhance the regulatory interaction of cTnC with cTnI (apparent Ca^2+^ sensitization) or inhibit (apparent Ca^2+^ desensitization) by either orienting cTnC to accept the switch peptide or sequester the switch peptide from cTnC by altering the interaction of cTnI with actin (**point 8**—actin and cTnC can be thought of as competitive binders for the switch peptide of cTnI) [37].

In a resting muscle, the switch peptide and inhibitory peptide of cTnI’s C-terminal domain are bound to actin physically pinning cardiac tropomyosin (cTm) overtop the strong binding sites of myosin on actin (steric blocking mechanism). If cTnC can more easily remove the inhibitory positioning of cTnI on actin, then this would appear as an apparent increase in Ca^2+^ sensitivity of force development and desensitization if this is harder to achieve. CTm flexibility and mobility itself can also be reflected as an apparent shift in Ca^2+^ sensitivity (**point 9**) [51,71,72,73,74,75]. Thus, even if all the functionalities of the cTn complex are operating as expected, cTm may be more or less cooperative in moving out of the way for myosin. An easily movable cTm could add to the apparent sensitization to Ca^2+^ and desensitization if harder to “rock and roll”. Ultimately, strong binding and force producing-binding of myosin to actin leads to an apparent Ca^2+^ sensitization of cTnC (**point 10**) [37,76]. Anything that would make it more difficult for myosin to strongly bind actin will be reflected as an apparent decrease in Ca^2+^ sensitivity, whereas enhancing strong myosin binding to actin will be reflected as a Ca^2+^ sensitization, possibly by locking cTm in the open state and favoring cTnC binding of cTnI over actin [49,77]. If these longer-lived cross-bridges become “dead-heads” and do not detach, then that region of the thin filament can be permanently activated without Ca^2+^.

As can occur in disease, anything that makes the contractile apparatus structurally disarrayed can influence any number of these steps and modulate the apparent Ca^2+^ sensitivity of the contractile apparatus. Finally, the cellular environment, such as pH and free Mg^2+^ concentration, can also affect the regulatory N-domain of cTnC, making it harder for Ca^2+^ to bind and, in essence, lead to an apparent Ca^2+^ desensitization [32,78]. Thus, there are a plethora of molecular mechanisms that can influence both the intrinsic and apparent Ca^2+^ sensitivity of cardiac muscle contraction via the known protein function of cTnC. Ultimately, any Ca^2+^ sensitivity measurement will be relative as Ca^2+^ sensitivity is highly dependent upon (1) the system being measured (isolated cTnC, cTn complex, ATPase, motility, force, etc.); (2) the biophysical approach/tools employed (fluorescence, microscopy, force transducer, chemistry, etc.); (3) experimental conditions (pH, buffer composition, temperature, Ca^2+^ chelator(s), species, etc.); and (4) experience and expertise of the investigative team. It is no wonder different groups will arrive at different results, even with quality control measures in place, considering there is no standardization for any of the biophysical approaches commonly used in the field.

We generally assume the only way for a mutant protein to alter cardiac function is via its direct protein function within the sarcomere. Recent and unexpected discoveries suggest that cTn may have an unknown functionality in the nucleus independent of its sarcomeric regulation of contraction [26]. Surprisingly, in non-muscle cells, nuclear cTn has been observed, too, and may be involved in pathways leading to cancer [79]. Thus, cTnC may have an influence on health that has nothing to do with its sarcomeric function. Another unexplored possibility is the mishandling of cTnC’s mRNA. Recent advances in the specialization and localization of mRNA in the heart suggest that proteins are translated at the sites of their use, termed local or distributed protein synthesis [80]. It may be that cTnC mRNA processing, handling, lifetime, and/or localization are maligned by genetic mutations. Thus, there may be issues caused by cTnC mutations (as well as any protein mutation, for that matter) that influence how the sarcomere is made and/or maintained at the nucleotide level. We fully expect future discoveries to be made in these exciting fields of study that will shed more light on the normal physiology and pathophysiology of the heart.

## 6. How Might cTnC Mutations Lead to Disease?

An imbalance in Ca^2+^ sensitivity has been thought to drive cardiac remodeling and even arrhythmic burden [3,81,82,83]. Considering the Ca^2+^ sensor (cTnC) and Ca^2+^ signal work hand in hand to set the contractile performance of the heart, it is not hard to see that an obvious way for the heart to counteract too much or not enough activation via cTnC would be to modulate the Ca^2+^ signal itself to compensate for the change in Ca^2+^ sensitivity and subsequent change in Ca^2+^ buffering by cTnC [84,85]. Considering force is not the only Ca^2+^-sensitive function or output in the heart, it is easy to see that if the heart has to readjust its Ca^2+^ signal to meet the demands of the body, that this could drastically alter all sorts of ion channels, signaling cascades and cell growth. To maintain a particular “Goldilocks” zone of force production in the face of altered Ca^2+^ sensitivity, the “aberrant” Ca^2+^ signal will also adversely alter Ca^2+^-dependent kinase activities (i.e., CaMKII) and phosphatases (i.e., calcineurin), and CaM activation in general (since CaM influences a large number of cellular functions) that are thought to drive cardiac remodeling, energetics, and electrical imbalances [81]. Thus, it is not difficult to understand that if the Ca^2+^ sensitivity is always out of line with normal function there will be negative consequences if only changes in Ca^2+^ are the counterbalance. However, not all cardiomyopathies, even when associated with myofilament mutations, appear to have an apparent shift in Ca^2+^ sensitivity, nor are all apparent shifts in Ca^2+^ sensitivity met with altered Ca^2+^ signaling (several described below for cTnC). This has raised concerns that Ca^2+^ sensitivity itself may not be the most important parameter that “causes” disease. We think this issue may revolve around how Ca^2+^ sensitivity is altered (there are several different ways this can be achieved, as described above). Some mechanisms that alter Ca^2+^ sensitivity may have no ill effect, while others can be readily compensated, whereas, in others, the compensation itself drives disease.

In some cases, the change in Ca^2+^ sensitivity may be conditional such that under normal conditions, everything seems fine, but under adrenergic stress, or deep relaxation, the myofilaments cannot re-tune to “shift gears” [36,86]. It is very common for the response of the myofilaments to be blunted or even blocked from being altered by the various post-translational modifications that occur on the heart in day-to-day fluctuations and over a lifetime of the individual. Thus, the altered Ca^2+^ sensitivity is not necessarily present under all cellular or physiological conditions. Furthermore, Ca^2+^ sensitivity is a steady-state parameter, but the beating heart is never in a steady state. Changes in Ca^2+^ sensitivity can only be inferred in vivo via changes in muscle contractility. It has been suggested that the problem is not in the change in the Ca^2+^ sensitivity itself but in the kinetic ramifications of the change in Ca^2+^ sensitivity [84,86]. We and others have observed that altering the Ca^2+^ sensitivity of cTnC can have a substantial impact on the rates of both contraction (isometric and isotonic) and relaxation (isometric and isotonic) [38,39,40]. Thus, there is more than one way for cTnC to have a negative (as well as potentially positive) impact on cardiac performance or cardiovascular health. As discussed above, in some instances, disease may be triggered by non-sarcomeric or even pre-protein effects. It is clear from the review of the human literature and current cTnC mutant mouse models that we are far from resolving the key drivers of cardiomyopathies even when we only focus on the simple, small protein cTnC.

## 7. Known HCM-, RCM-, DCM, and LVNC-Associated Mutations in Human cTnC

**Figure 2:**
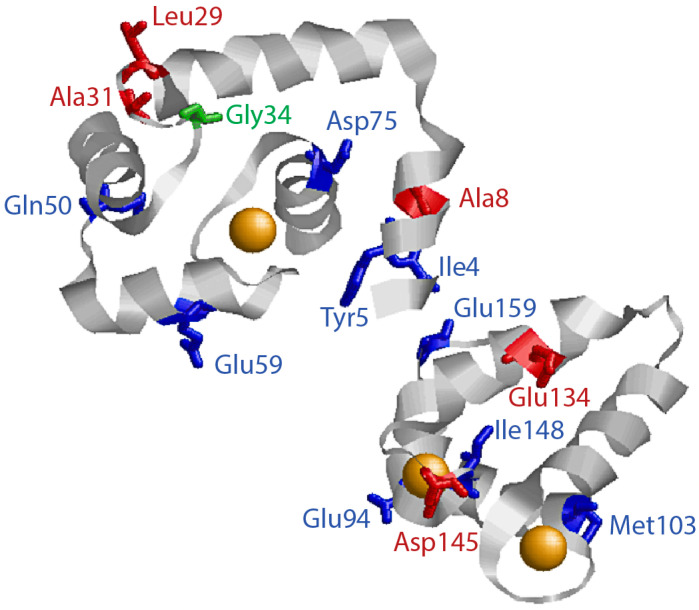
HCM, DCM, and LVNC Associated Mutations in cTnC. The figure shows the structure of human cTnC, highlighting amino acid variants linked to HCM (red), DCM (blue), or LVNC (green) that have been published in peer-reviewed journals. The figure was generated using RasWin Molecular Graphics (Windows Version 2.7.5.2) [96] utilizing the human cTnC structure from PDB 4y99. C84 is not shown due to missing this part of the central helix in the crystal structure.

The first mutation linking cTnC to cardiomyopathy, L29Q, was discovered over 20 years ago [87]. Since then, a number of additional mutations linking cTnC to HCM, RCM, DCM, and LVNC have been discovered [54]. Figure 2 schematically depicts where each of the discussed human-associated cTnC mutants occur within the structure of cTnC. The known HCM-, RCM-, DCM- or LVNC-linked mutations in cTnC appear to be relatively rare but might not be as rare as originally believed as each day the list of potentially disease-causing mutations in cTnC grows. While the vast majority of human patients are heterozygous for any given cTnC mutation, there have been several cases of parents each passing on either a different or the same cTnC mutation to their offspring with catastrophic consequences [54]. Interestingly, individuals carrying likely pathogenic mutations in TNNC1 typically present at a younger age of diagnosis, have higher rates of potentially fatal events, and a worse prognosis for survival than individuals carrying mutations in TNNT2 and TNNI3 [88], underscoring the high importance of cTnC in the regulation of cardiac contractility. HCM-linked mutations in cTnC for which some clinical/biochemical/physiological information is available include A8V, L29Q, A31S, C84Y, E134D, and D145E, while compound heterozygosity for A8V and D145E mutations was linked to RCM [89]. DCM-linked mutations include I4M, Y5H, Q50R, E59D/D75Y, E94V, M103I, D145E, I148V, and G159D [21,54,90], while LVNC-linked mutations include G34S [91]. To date, two genetically engineered mouse models expressing cTnC mutations linked to disease in humans (A8V and C84Y) have been created [92,93]. It has been noticed that HCM- and RCM-linked mutations in regulatory thin filament proteins tend to sensitize myofilaments to Ca^2+^, while DCM-linked mutations tend to desensitize [94,95].

## 8. Mutations in cTnC Linked to HCM

The A8V mutation, located within the N-helix of cTnC, was identified from a large cohort of 1025 unrelated patients [97]. This mutation was found in a 34-year-old patient diagnosed with severe obstructive HCM [93]. The patient exhibited increased septal, left, and right ventricular wall thickness, decreased left ventricular systolic and diastolic dimensions, enlargement of the left atrium, and hyper-dynamic left ventricular systolic function [93]. While the A8V mutation did not increase the Ca^2+^ binding affinity of the regulatory N-domain of isolated cTnC, it enhanced the binding affinity of cTnC for the switch region of cTnI [55,98]. As a consequence of enhanced interactions with cTnI, the A8V mutation led to increased Ca^2+^ sensitivity of reconstituted thin filament, combined with a slower rate of Ca^2+^ dissociation in the absence or presence of myosin S1 [43,99]. The A8V mutation also led to an increase in the Ca^2+^ sensitivity of actomyosin ATPase and force development in cardiac muscle [43,99]. Thus, the A8V mutation has biochemical/physiological properties generally assumed to be typical of HCM-linked thin filament mutations (such as increased Ca^2+^ sensitivity), implicating it in a hypertrophic phenotype observed in the patient.

The L29Q mutation, located within the defunct first EF-hand of cTnC, was identified in a 60-year-old male patient with an onset of disease at 59 years of age [87]. The familial incidence of the mutation is unknown. There have been some discrepancies in the biochemical/physiological properties of this mutation reported by different groups. For instance, the L29Q mutation was shown to result in a modest increase in the Ca^2+^ sensitivity of the N-domain of isolated cTnC [45] and a decrease in the Ca^2+^ sensitivity of reconstituted thin filaments [100]. However, a different group reported that L29Q produced a small but insignificant increase in the Ca^2+^ sensitivity of reconstituted thin filaments [101]. L29Q was observed to modestly increase the Ca^2+^ sensitivity of force development in skinned mouse cardiac myocytes [45], with the Ca^2+^ sensitizing effect being more pronounced at shorter sarcomere lengths [100]. However, other groups reported that L29Q did not alter the Ca^2+^ sensitivity of force development or interfere with the PKA-induced decrease in the Ca^2+^ sensitivity of force development in skinned mouse cardiac fibers [102] or porcine cardiac muscle [101]. Yet another group reported that L29Q decreased the Ca^2+^ sensitivity of force development in rat cardiac muscle [103]. Due to discrepancies in reported results, it is difficult to draw conclusions about the molecular mechanisms of the L29Q mutation, but it has generally been assumed to revolve around some sort of aberrant Ca^2+^ sensitivity.

Mutation A31S, located within the defunct first EF hand, was identified in a pediatric patient with multiple episodes of ventricular fibrillation and aborted sudden cardiac death [104]. A31S appears to be a de novo mutation, as it was not found in either of the patient’s parents. The A31S mutation led to an increase in Ca^2+^ sensitivity of isolated cTnC, cTn complex, and thin filaments in the absence or presence of myosin S1 [104]. The A31S mutation also led to an increase in the Ca^2+^ sensitivity of actomyosin ATPase and force development [104]. Thus, the A31S mutation has biochemical/physiological properties assumed to be typical of HCM-linked thin filament mutations (such as increased Ca^2+^ sensitivity), implicating it in the hypertrophic phenotype observed in the patient.

Mutation C84Y, located at the end of the D-helix, was found in a 17-year-old male diagnosed with HCM at 8 years old [97]. The patient with the C84Y mutation did not present with a family history of HCM, suggesting it was a de novo mutation. Biochemical studies suggested that the C84Y mutation shifted the equilibrium of the N-domain toward a more “open” state [92] that presumably favors switch peptide binding. Consistent with this model of cTnC activation, the C84Y mutation led to an increase in the Ca^2+^ sensitivity of actomyosin ATPase [98] and force recovery in skinned porcine fibers [97]. Thus, the C84Y mutation has biochemical/physiological properties assumed to be typical of HCM-linked mutations.

The E134D mutation, located in the G-helix, was found in a female patient, diagnosed with HCM at 16 years old, who died at 22 years old of unspecified cause [97]. No familial incidence is available for this mutation. The C-terminal E134D mutation did not affect either the Ca^2+^ sensitivity of reconstituted thin filaments or the rate of Ca^2+^ dissociation [99]. E134D also had no significant effect on the Ca^2+^ sensitivity of acto-myosin ATPase [99] or force development [97]. Based solely on no observed changes in Ca^2+^ sensitivity, this mutation has been suggested to be a benign polymorphism [95], or it could lead to the development of HCM through yet unexplored mechanisms (see Figure 1 and discussed above). A deeper biophysical evaluation and a transgenic mouse model expressing this mutation would be helpful in answering these questions.

The D145E mutation, located within the fourth Ca^2+^ binding loop, was found in a 58-year-old male with a positive family history of HCM [97]. The D145E mutation led to a drastic reduction in the Ca^2+^ affinity of the C-terminal domain of cTnC, likely due to its location in a Ca^2+^ chelating position [105]. The D145E mutation also led to an ~eight-fold reduction in the affinity of cTnC for the regulatory region of cTnI at low physiological levels of Ca^2+^ [105]. The D145E mutation resulted in increased Ca^2+^ sensitivity of reconstituted thin filaments with a slower rate of Ca^2+^ dissociation from reconstituted thin filaments in the presence of myosin S1 [43,98]. The D145E mutation also led to substantial increases in the maximal actomyosin ATPase activity and force development in porcine cardiac muscle strips [43,97]. Interestingly, the D145E mutation was also found in a patient with DCM [106,107]. However, the DCM patient with the D145E mutation in cTnC also had a myosin-binding protein C mutation, which potentially contributed to the observed DCM phenotype [107]. Overall, the D145E mutation appears to have biochemical/physiological properties assumed to be typical of HCM-linked mutations.

## 9. Mutations in cTnC Linked to RCM

In a case of early onset RCM, two siblings inherited the A8V mutation from their father and the D145E mutation from their mother [89]. Family members carrying either A8V or D145E mutations alone appeared to be unaffected (unlike the cases described above), suggesting these mutations need an additional trigger or more time to cause morbidity or mortality. In fact, heterozygous A8VcTnC knock-in mice take substantially longer to develop hypertrophy than homozygous mice [93]. However, the two siblings who inherited both mutations from their parents developed restrictive cardiomyopathy with septal hypertrophy and died within the first year of life [89]. As mentioned above, it is generally assumed that both HCM and RCM are caused by Ca^2+^ sensitization, with greater sensitization resulting in RCM [108]. In the case of siblings with early onset RCM, the Ca^2+^ sensitivity of force development is expected to be higher than in either of their parents since A8V and D145E are both Ca^2+^ sensitizing mutations.

## 10. Mutations in cTnC Linked to DCM

Mutation I4M, located within the N-helix, was identified in pediatric patients with severe DCM [57]. Biochemical studies revealed that the I4McTnC mutant displayed tighter binding to cTnT, suggesting that alterations in cTnC-cTnT binding could compromise contractile function triggering hypertrophic remodeling [57]. Porcine fibers reconstituted with I4McTnC exhibited modestly reduced Ca^2+^ sensitivity of force development [57]. This finding seems to support the generalization that decreased Ca^2+^ sensitivity leads to DCM. However, more studies are needed to evaluate the biochemical and physiological properties of this mutation.

Mutation Y5H, located within the N-helix, was identified from a cohort of patients with idiopathic DCM [107]. The Y5H mutation was found in a boy who was hospitalized with heart failure at two weeks old and later required a heart transplant at 15 years old. However, the patient carrying Y5HcTnC mutation also had a mutation in the β-myosin heavy chain, complicating the attribution of phenotype. The Y5H mutation resulted in reduced Ca^2+^ sensitivity of force development in porcine-skinned fibers and diminished effects of PKA phosphorylation on force development [106]. Thus, Y5H mutation has biochemical and physiological properties assumed to be typical of DCM-linked thin filament mutations.

Mutation Q50R, located between the B-and C-helices, was identified in a child diagnosed with DCM at 16 months old from a family with extremely variable expression of the disease [109]. While the child’s mother developed peripartum cardiomyopathy at 30 years of age, several family members in their sixties were diagnosed with mild DCM after screening [109]. The Q50R led to an increase in the Ca^2+^ sensitivity of isolated cTnC [78], but its effects on Ca^2+^ binding properties of cTnC incorporated into thin filaments or on Ca^2+^ sensitivity of force development remain unknown. Since isolated cTnC is not a good predictor of physiological outcome [33], more studies are needed to evaluate the biochemical and physiological properties of Q50R mutation.

A double mutation E59D/D75Y was identified in a patient with idiopathic DCM [110]. The E59D mutation is located within the C-helix, while the D75Y mutation is a non-chelating residue within the Ca^2+^ binding loop in site II. The family history of the patient was not available. The double E59D/D75Y mutation resulted in reduced Ca^2+^ sensitivity of isolated cTnC, the cTnC-cTnI complex, the cTn complex, and reconstituted thin filaments [111]. The E59D/D75Y mutation also led to reduced Ca^2+^ sensitivity in tension development in skinned cardiac fibers and to a reduction in maximal force recovery [111]. Experimental results suggested that the D75Y mutation was responsible for the Ca^2+^ sensitivity changes, but the E59D mutation enhanced the effects of D75Y with both E59D and D75Y required to significantly lower the maximal force development [111]. Intact rat ventricular myocytes expressing the D75Y (but not E59D) mutation exhibited impaired contractility, despite having normal Ca^2+^ transients [110]. Thus, the E59D/D75Y mutation has biochemical and physiological properties assumed to be typical of DCM-linked thin filament mutations (such as decreased Ca^2+^ sensitivity), implicating it in the dilated phenotype observed in a patient.

Mutation E94V, located at the beginning of the E-helix, was found in a 20-year-old male patient who started having decreased exercise tolerance at 13 years old [112]. At 16 years old, this patient was found to have a dilated left ventricle and ejection fraction of only 15%, requiring a heart transplant [112]. No biochemical data is available for the E94VcTnC mutant. However, our group showed that conserved charged residues in the central helix of cTnC, including E94, play a crucial role in the proper regulatory function of cTnC [61]. Replacement of charged central helix residues (including E94) with a neutral Ala resulted in reduced Ca^2+^ sensitivity of the cTn complex and reconstituted thin filaments; and desensitization of actomyosin ATPase to Ca^2+^ [61]. These changes were likely due to the loss of electrostatic interactions between the acidic residues in the central helix of cTnC and basic residues within the regulatory region of cTnI. Thus, the E94V mutation is predicted to result in reduced Ca^2+^ sensitivity of force development due to lower Ca^2+^ sensitivity of cTnC reconstituted into cTn complex and thin filaments. More studies are needed to evaluate the biochemical and physiological properties of this mutation.

Mutation M103I, located in the E-helix, was identified from a cohort of patients with idiopathic DCM [107]. The M103I mutation was found in a female diagnosed with DCM at 47 years old, who required an implantable cardiac defibrillator and pacemaker after cardiac arrest at 49 years of age. The patient had a sibling also diagnosed with DCM, suggesting M103I mutation segregated with the disease. The cTnC mutant carrying the M103I mutation led to reduced Ca^2+^ sensitivity of force development in porcine-skinned fibers [106]. In addition, the M103I mutation diminished the effects of PKA phosphorylation on force development [106]. Thus, the M103I mutation has biochemical and physiological properties assumed to be typical of DCM-linked thin filament mutations.

Mutation D132N is located in the G-helix and by itself has not been linked to DCM. Two siblings who inherited the D132N mutation from their father, and the D145E mutation from their mother, developed severe early-onset DCM [113]. Both parents were asymptomatic in their early forties and showed no sign of cardiovascular disease. One of the siblings died at 14 months of age before finding a heart donor. Her brother required cardiac transplantation at 13 months of age. A combination of 50% D132NcTnC with 50% wild-type cTnC led to a decrease in the Ca^2+^ sensitivity of force development, while the combination of 50% of D145EcTnC with 50% of wild-type cTnC led to an increase in the Ca^2+^ sensitivity of force development in skinned porcine fibers. When 50% of D132NcTnC was combined with 50% of D145EcTnC, the Ca^2+^ sensitivity of force development was not significantly different from that of 100% of wild-type cTnC. Therefore, the combination of D132NcTnC with D145EcTnC normalized the effects of each mutation on the Ca^2+^ sensitivity of force development by skinned porcine fibers [113]. Thus, biochemical/physiological studies failed to provide a molecular mechanism responsible for the severity of the phenotype associated with the presence of both mutations, assuming a change in Ca^2+^ sensitivity is the only driving factor of disease. In our attempts to engineer cTnC to counteract disease-associated mutations in the other cTn subunits, we noticed that some cTnC mutations could “correct” the aberrant Ca^2+^ sensitivity but not the aberrant Ca^2+^ exchange kinetics, whereas other cTnC mutations could correct the aberrant kinetics, but not the sensitivity [30]. Thus, it would be beneficial to know the combined effect of the D132N and D145E mutations on Ca^2+^ exchange rates with the thin filament, in addition to their effect on the steady-state properties. Further biophysical studies and animal models would be beneficial in determining the molecular mechanism responsible for triggering severe dilated phenotype in the two siblings.

Mutation I148V, a non-chelating residue in the Ca^2+^ binding loop in site IV, was identified from a cohort of patients with idiopathic DCM [107]. The I148V mutation was found in a female diagnosed with DCM at 40 years old. The I148V mutation had no effect on the Ca^2+^ sensitivity of force development but diminished the effects of PKA phosphorylation on force development [106]. Thus, the aberrant Ca^2+^ sensitivity in this case appears conditional and may not be present under basal conditions. It has been hypothesized that suppression of lusitropy can trigger cardiomyopathies [86], so it could be the molecular mechanism by which the I148V mutation exerts its pathogenic effects.

The G159D mutation, located in the C-terminus of cTnC, was identified in a family with an extensive history of DCM [114]. G159D was associated with severe disease with complete penetrance, with a number of family members requiring cardiac transplantation or experiencing premature death [114]. The youngest G159D patient received a heart transplant at three years old [115]. As with L29Q, this C-domain mutation has been studied by multiple groups, with some discrepancies in the reported results. While the G159D mutation did not affect the Ca^2+^ sensitivity of the cTn complex [116], it led to a reduction in the Ca^2+^ affinity of reconstituted thin filaments [101,117]. It was reported that the G159D mutation did not affect Ca^2+^ sensitivity of force development in skinned rat cardiac trabeculae but blunted the extent of Ca^2+^ desensitization in force development induced by cTnI phosphorylation [116]. On the other hand, a different study reported that G159D led to a modest decrease in Ca^2+^ sensitivity of myofibrillar ATPase and force development in skinned cardiac porcine fibers [101]. However, the Ca^2+^ sensitivity of force development in skinned ventricular myocytes from the three-year-old patient carrying the G159D mutation was higher than that from a non-failing donor heart [118]. The study also found that the G159D mutation blunted the change in the Ca^2+^ sensitivity of the sliding speed in thin filaments induced by cTnI dephosphorylation [118]. Given discrepancies in the reported results, it is difficult to make conclusions regarding the molecular mechanism(s) of this mutation, but the change in Ca^2+^ sensitivity and/or kinetics may be conditional during times of β-adrenergic stimulation.

## 11. Mutations in cTnC Linked to LVNC

Mutation G34S, located within the defunct first EF hand, was discovered in a newborn diagnosed with LVNC, who required a heart transplant shortly after diagnosis [91]. This patient’s heart was characterized by dilated ventricles with impaired systolic and diastolic function, hypertrabeculation, and fibrosis. The G34S mutation induced a disturbance in the thin filament morphology, with thin filaments appearing partially fragmented and bundled. Skinned cardiac myocytes isolated from the explanted heart of the patient carrying the G34S mutation exhibited higher Ca^2+^ sensitivity of force development with reduced maximal force compared to the donor heart. More studies involving G34S and other LVNC-linked mutations in thin filament proteins are needed to determine whether these mutations share common characteristics or are personalized.

## 12. Summary of the Data on Human Patients

More and more mutations are being discovered in cTnC with each passing day that are being linked to each of the classical and newly termed cardiomyopathies [54]. Unfortunately, there is typically insufficient clinical data, particularly longitudinally, to fully understand disease presentation other than the generalized cardiomyopathic categorization. This is particularly problematic for de novo mutations since there are no family histories available [57]. However, even when the same mutation has been found in several different people, very few of the mutations have high penetrance. Although many of the human cTnC mutations appear to influence the Ca^2+^ sensitivity of several different biophysical preparations in a predictable way, it is hard to understand how the change in Ca^2+^ sensitivity alone is the key driving factor of disease. Clearly, more information, from the human studies, biophysical characterization, and animal models is required for a clearer understanding of disease drivers.

## 13. Genetically Engineered Mouse Models

Unfortunately, the family incidence of the disease is unknown for many of the reported HCM- and DCM-linked cTnC mutations [54]. Some of the mutations, especially the ones leading to early onset/severe disease, appear to be de novo [54]. In some cases, mutations are discovered in more than one gene, complicating the attribution of phenotype. Animal models can provide crucial evidence of whether these mutations are pathogenic variants or benign polymorphisms. Developing animal models that accurately recapitulate features of cardiomyopathies should enhance our understanding of pathophysiological mechanisms and lead to the development of novel treatment strategies. Mouse models are highly valuable and widely used tools to study human diseases, including diseases affecting the cardiovascular system [119,120,121]. To date, five genetically engineered mouse models expressing cTnC mutants have been created, as described below.

The A8VcTnC knock-in mouse model was created via gene targeting technology in order to test whether TNNC1 is an HCM-susceptibility gene [93]. Both heterozygous and homozygous animals were viable, with heterozygous animals generally exhibiting less of an effect compared to homozygous animals [93]. Homozygous A8V knock-in mice exhibited significantly increased Ca^2+^ sensitivity of force development in skinned papillary fibers (ΔpCa_50_ = 0.4–0.5) while decreasing cooperativity [122]. In addition, the A8V mutation resulted in the acceleration of cross-bridge kinetics in skinned fibers isolated from homozygous mice [122]. The perturbation in cross-bridge kinetics could have been caused by the reduction in phosphorylation of multiple Thr residues in the rod domain of α-MHC [122]. In addition, skinned cardiac fibers isolated from homozygous knock-in mice displayed faster rates of tension redevelopment at all levels of Ca^2+^ activation [123]. Skinned mutant fibers also displayed increased myofilament spacing at sub-maximal levels of Ca^2+^ activation, suggesting that transient expansion in lattice spacing during Ca^2+^ activation contributes to an increase in the rate of cross-bridge cycling [123]. Furthermore, skinned fibers isolated from homozygous knock-in mice had a significantly slower rate of relaxation [93]. Echocardiographic measurements demonstrated that as early as three months of age, homozygous knock-in mice exhibited reduced end-diastolic volume, end-systolic volume, and left ventricular dimensions [93], with knock-in female mice exhibiting a statistically significant increase in isovolumetric contraction time compared to knock-in males [124]. At three and nine months of age, homozygous A8V knock-in mice had higher ejection fraction, consistent with hyperdynamic left-ventricular systolic function observed in the human patient [93]. Histopathological analysis demonstrated papillary muscle hypertrophy and increased interstitial fibrosis in the hearts of sixteen- to eighteen-month-old mice [93]. In twelve- to thirteen-month-old knock-in mice, heart-weight-to-body-weight ratios were significantly reduced, while left atrial mass was dramatically increased, suggesting adaptive remodeling [93]. Intact myocytes isolated from homozygous mice exhibited reduced diastolic sarcomere length, increased contractile percentage, slower shortening and re-lengthening, and prolonged Ca^2+^ transients [93]. Thus, the mouse model provided evidence that A8V mutation in cTnC could be responsible for the development of HCM in a patient. In addition, the authors hypothesized that the A8V mutations cause problems due to incomplete relaxation during diastole, with the system trying to compensate for the impaired diastolic function by enhanced adrenergic and other cellular mechanisms. This could cause over-working of the heart, leading to reduced phosphorylation of the rod domain of α-MHC, and eventually result in hypertrophic phenotype. However, although the mice’s hearts possessed biophysical properties and remodeling indicative of HCM, there was no evidence to suggest this led to an increase in morbidity or mortality.

The C84YcTnC knock-in mouse model was generated via gene-targeting technology to determine whether the HCM-linked C84Y mutation recapitulates HCM phenotype in mice [92]. Homozygous mice were not obtained, suggesting the C84Y mutation in both alleles led to embryonic lethality. Thus, only heterozygous mice were studied. Papillary fiber bundles isolated from six-month-old C84Y knock-in mice demonstrated increased Ca^2+^ sensitivity of force development (ΔpCa_50_ = 0.16). In addition, at sub-maximal Ca^2+^, papillary muscle bundles isolated from knock-in mice exhibited a slower rate of tension redevelopment. Computational modeling suggested that C84Y mutation resulted in ~two-fold faster rate of cross-bridge detachment and two-fold slower rate of cross-bridge attachment. Echocardiographic experiments showed that by three months of age, C84Y knock-in mice already displayed features consistent with HCM, such as decreased systolic/diastolic dimensions, reduced stroke volume, and cardiac output. At three months of age, knock-in mice also exhibited decreased mitral valve early peak flow velocity/atrial peak flow velocity. At six months of age, knock-in mice exhibited significantly increased left posterior systolic and diastolic wall thickness, and by eight to nine months of age, knock-in mice exhibited increase in myocardial fibrosis. Thus, the C84Y mutation in mice was able to recapitulate the hypertrophic phenotype. Again though, there is no evidence that the HCM-related changes in the mice’s hearts caused increased morbidity or mortality.

While the A8V and C84Y mutation recapitulated hallmark features of HCM, transgenic mice expressing the potent Ca^2+^ sensitizing L48Q mutation (an engineered mutation not discovered in humans [32] conditionally expressed in the heart did not develop a hypertrophic phenotype [84]. L48Q mutation was found to substantially increase the Ca^2+^ sensitivity of isolated cTnC, the cTn complex, and reconstituted thin filaments [32,33]. Cardiac myocytes isolated from L48Q transgenic mice had increased fractional shortening and prolonged relaxation, consistent with higher myofilament Ca^2+^ sensitivity [84]. The L48Q did not affect the twitch peak tension but significantly prolonged relaxation times in trabeculae isolated from the hearts of transgenic mice [40]. However, other studies have shown that slowed relaxation was not observed in isolated ventricular myocytes from mice and rats (with or without myocardial infarction) expressing L48QcTnC via adeno or adeno-associated virus gene transfer [31,125]. In fact, relaxation was actually improved by expressing L48QcTnC in the myocytes from infarcted rat hearts [125]. In addition, relaxation was not compromised when measured in vivo in mice (with or without myocardial infarction) expressing L48QcTnC via adeno-associated virus gene transfer [31]. The observed discrepancies in the effect of L48Q on relaxation could be due to higher levels of L48QcTnC expression in transgenic mice versus virally induced expression. Interestingly, L48Q transgenic mice did not exhibit any changes in cardiac growth or chamber dimensions up to one year of age [84], similar to mice with in vivo viral transduction of L48QcTnC [31]. The study suggested that despite failing to develop a hypertrophic phenotype under normal mouse husbandry conditions, L48Q transgenic mice were predisposed to hypertrophic growth, as administration of β-receptor antagonist metoprolol for three months led to increases in septal wall thickness and cardiac mass in these mice [84].

Our group showed that expression of L48QcTnC in mouse myocardium via adeno-associated virus gene transfer was actually beneficial after myocardial infarction [31]. In addition, a recent study utilized L48Q transgenic mice to treat a genetic form of DCM [126]. In that case, transgenic mice expressing L48QTnC were crossed with transgenic mice expressing the DCM-linked D230NcTm mutation [126]. D230NcTm transgenic mice develop significant systolic dysfunction and dilation by two months of age, with reduced Ca^2+^ sensitivity of force development. In contrast, cardiac muscle isolated from double transgenic mice (expressing both L48QcTnC and D230NcTm) had Ca^2+^ sensitivity of force development similar to that of wild-type mice. While echocardiographic parameters of D230NcTm mice indicated progressively worsening dilated phenotype (from ages of two to five months), echocardiographic parameters of double transgenic mice were similar to that of wild-type mice over the same time period. Thus, the expression of Ca^2+^ sensitizing L48QcTnC in the heart of D230NcTm transgenic mice prevented the development of dilated phenotype [126]. Thus, L48QcTnC or other cTnC mutants with desired properties [29,30] could potentially be used to treat cardiovascular diseases.

The A8VcTnC and C84YcTnC mouse models were created to evaluate whether the incorporation of known HCM-linked mutations found in human patients, into mouse myocardium could recapitulate the hypertrophic phenotype. A different approach was taken when creating a mouse model of DCM [127]. Would a Ca^2+^ desensitizing cTnC mutation not linked to DCM in human patients result in dilated phenotype in mice? The D73N mutation, not discovered in humans, was rationally engineered to decrease the Ca^2+^ sensitivity of the regulatory N-domain of cTnC [127]. Biochemical studies revealed that the D73N mutation resulted in substantially lower Ca^2+^ sensitivity (ΔpCa_50_ = −0.55) of reconstituted thin filaments due to ~three-fold faster rate of Ca^2+^ dissociation. Potentially as a consequence of reduced Ca^2+^ sensitivity, the D73N mutation blunted the extent of thin filament Ca^2+^ desensitization induced by cTnI pseudo-phosphorylation [127]. The D73N mutation was then knocked into the mouse endogenous TNNC1 gene via gene targeting technology. None of the homozygous mice survived longer than one day, so only heterozygous mice were evaluated. The amount of D73N transcript in the left ventricle of heterozygous knock-in mice was estimated at ~31% of the total cTnC transcript. Heterozygous mice were born with an expected Mendelian frequency and were viable and fertile. As expected, the Ca^2+^ sensitivity of force development of skinned ventricular trabeculae was substantially reduced in heterozygous knock-in mice (ΔpCa_50_ = −0.23).

D73N knock-in mice did not display any obvious differences in body weight, appearance, or activity levels compared to their wild-type littermates. However, all knock-in mice died by nineteen weeks of age, with a median survival of twelve weeks. Histological analysis of the hearts revealed a gradual increase in heart size and dilation of the left ventricular chamber over time. By four weeks of age, D73N knock-in mice already had increased heart-weight-to-body-weight ratios. At the time of death, the D73N knock-in mice had heart-weight-to-body-weight ratios four to five times larger than their wild-type litter mates. The D73N mutation also resulted in a small but statistically significant increase in myocardial fibrosis. Echocardiographic analysis revealed that knock-in mice developed hallmark features of early-onset DCM, with substantially reduced ejection fraction, enlarged left ventricular dimensions with thinned left ventricular walls, and sudden death with 100% penetrance. Mice also exhibited electrophysiological abnormalities, such as significantly prolonged QRS and QT intervals. Ventricular myocytes isolated from knock-in mice had significantly longer resting sarcomere length, consistent with reduced myofilament Ca^2+^ sensitivity. In addition, ventricular myocytes isolated from knock-in mice were not responsive to β-adrenergic stimulation. A similar dilated phenotype with increased mortality was observed when the D73N mutation was introduced into mouse myocardium via adeno associated gene transfer [31]. Thus, desensitizing cTnC to Ca^2+^ not only leads to the hallmarks of DCM but early mortality too.

Consistent with results observed for D73N knock-in mice, transgenic mice conditionally expressing a Ca^2+^ desensitizing cTnC with the I61Q mutation (an engineered cTnC not found in humans [128] specifically in the heart exhibited early mortality (with no transgenic mice surviving beyond eight months) and increased heart-weight-to-body-weight ratios [84]. Myocytes isolated from I61Q transgenic mice had significantly longer sarcomere lengths, consistent with reduced myofilament Ca^2+^ sensitivity [84]. In addition, peak twitch tension in trabeculae isolated from I61Q transgenic mice was reduced by ~60%, while relaxation times were not affected [40]. Echocardiography demonstrated that by six-eight weeks of age, I61Q transgenic mice developed a dilated phenotype characterized by substantially reduced fractional shortening, increased left ventricular diastolic chamber dimensions, and decreased septal wall thickness [84]. Thus, results from D73NcTnC and I61QcTnC mouse models support the idea that Ca^2+^ desensitization of cardiac muscle serves as a trigger in the development of the dilated phenotype.

Since cTnC is also expressed in slow skeletal muscle, D73N knock-in mice were used to examine the effect of desensitizing cTnC to Ca^2+^ on the function of skeletal muscle [35]. The D73N mutation resulted in a substantial decrease in the Ca^2+^ sensitivity of force development in slow-twitch fibers isolated from the soleus muscle of mice (ΔpCa_50_ = −0.36) but not from fast-twitch fibers that express fast skeletal TnC and not cTnC [35]. The D73N mutation led to a rightward shift in the force versus stimulation frequency relationship and reduced resistance to fatigue in intact soleus muscle [35]. The D73N mutation resulted in a significant reduction in the cross-sectional area of slow-twitch fibers without affecting the fiber type composition of the soleus muscle [35]. The D73N mutation also resulted in significantly faster kinetics of isometric twitches and tetani in mouse soleus muscle [35]. Interestingly, of the three HCM-linked mutations (A8V, C84Y, and D145E) that sensitized cardiac muscle to Ca^2+^, only C84Y sensitized slow skeletal muscle to Ca^2+^ [129]. Thus, some cTnC mutations can also lead to profound alterations in the function of slow skeletal muscle, which should be taken into account when treating human patients with cTnC mutations.

## 14. Summary of the Data in Genetically Engineered Mice

In conclusion, five genetically engineered mouse models expressing cTnC mutants have been created to date. Mice expressing either of the HCM-linked Ca^2+^ sensitizing mutations (A8V and C84Y) were able to recapitulate the hypertrophic phenotype observed in human patients carrying these mutations, yet without increased morbidity or mortality. On the other hand, mice expressing the Ca^2+^ sensitizing mutation L48Q (not linked to disease in human patients) failed to develop hallmark hypertrophic phenotypes under normal mouse husbandry conditions. In fact, the L48Q mutation was beneficial in treating DCM in D230NcTm transgenic mice [126] and, more generally, myocardial infarction via a gene therapy approach [31]. Thus, Ca^2+^ sensitization by itself might not be sufficient to trigger the development of the hypertrophic phenotype unless combined with some other insult or factor, such as being male or female [124]. Mice carrying either of the two Ca^2+^ desensitizing mutations (D73N and I61Q), not linked to disease in humans, developed a severe dilated phenotype with a shortened lifespan. Whether Ca^2+^ desensitization in itself always triggers dilation will involve more studies, including additional animal models. Regardless, the animal models are consistent with the human findings that conditions which lead to DCM (Ca^2+^ desensitization) are more detrimental to health and lifespan than those that show hallmarks of HCM (Ca^2+^ sensitization and ventricular hypertrophy). These findings suggest that there may be many more beneficial compensatory mechanisms for HCM than DCM or that Ca^2+^ sensitization itself is not a disease driver.

## 15. Conclusions

Clearly, more information, from the human studies, biophysical characterizations, and animal models, is required for a clearer understanding of disease drivers that lead to cardiomyopathies. The field may consider a way to standardize experimental tests or, at minimum, arrive upon a minimal set of experiments under specific conditions that should be run for all sarcomeric mutations. The unique use of an engineered cTnC that had been thoroughly characterized and genetically introduced into mice clearly demonstrates that Ca^2+^ sensitization in and of itself should not necessarily be considered a disease driver. This opens the door for small molecule [130,131] and protein engineering strategies to help boost impaired systolic function. On the other hand, the engineered cTnCs demonstrate that Ca^2+^ desensitization under basal conditions may be a driving factor for DCM. In addition to enhancing our knowledge of molecular mechanisms that trigger hypertrophy, dilation, morbidity, and mortality, these cardiomyopathy mouse models could be used to test novel treatment strategies for cardiovascular diseases.

## Data Availability

No new data were created or analyzed in this study. Data sharing is not applicable to this article.
